# The moderating role of emotion regulation on the relationship between sensitivity to punishment and aggressive behaviour

**DOI:** 10.1192/j.eurpsy.2022.1710

**Published:** 2022-09-01

**Authors:** A. Megías-Robles, R. Gómez-Leal, R. Cabello, M.J. Gutiérrez-Cobo, P. Fernández-Berrocal

**Affiliations:** 1 Faculty of Psychology. University of Málaga, Department Of Basic Psychology, Málaga, Spain; 2 Faculty of Psychology. University of Granada, Department Of Developmental And Educational Psychology, Granada, Spain; 3 Faculty of Psychology. University of Málaga, Department Of Developmental And Educational Psychology, Málaga, Spain

**Keywords:** Aggressive behaviour, sensitivity to punishment, emotional regulation

## Abstract

**Introduction:**

The role of sensitivity to punishment on aggression is controversial, both positive and negative relationships have been observed in previous literature.

**Objectives:**

The aim of this research was to clarify the role of sensitivity to punishment in different types of aggression and provide a better understanding of the influence of emotional regulation on this relationship.

**Methods:**

Two hundred and twenty-nine participants took part in the study (130 women; average age = 21.52 years). All of them were assessed for levels of verbal aggression, physical aggression, anger, and hostility (by Buss-Perry Aggression Questionnaire), levels of sensitivity to punishment (by SPSRQ–20), and emotional regulation ability (by MSCEIT).

**Results:**

A higher reactivity to punishment had a direct negative effect on physical and verbal aggression. However, a higher reactivity to punishment also showed a positive indirect effect on verbal and physical aggression through an increase in anger and hostility. In addition, ability in regulating emotions moderated the indirect effects of sensitivity to punishment on physical aggression.

**Conclusions:**

Our results suggest that sensitivity to punishment can act both as a protective factor and as a risk factor for aggression. This relationship depended on the type of aggression studied and the emotional regulation abilities. These findings can help to inform the design of programs aimed at reducing aggressive behaviour. This work was funded by Junta de Andalucía (projects: EMERGIA20_00056 and UMA18-FEDERJA-137) to Alberto Megías Robles.

**Disclosure:**

No significant relationships.

